# Exploring the role of organizational policies and procedures in promoting research utilization in registered nurses

**DOI:** 10.1186/1748-5908-2-17

**Published:** 2007-06-05

**Authors:** Janet E Squires, Donna Moralejo, Sandra M LeFort

**Affiliations:** 1 Knowledge Utilization Studies Program, Faculty of Nursing, University of Alberta, Edmonton, Alberta T6G 0P2, Canada; 2 School of Nursing, Memorial University of Newfoundland, St. John's, NL A1B 3V6, Canada

## Abstract

**Background:**

Policies and procedures (P&Ps) have been suggested as one possible strategy for moving research evidence into practice among nursing staff in hospitals. Research in the area of P&Ps is limited, however. This paper explores: 1) nurses' use of eight specific research-based practices (RBPs) and RBP overall, 2) nurses' use and understanding of P&Ps, and 3) the role of P&Ps in promoting research utilization.

**Methods:**

Staff nurses from the eight health regions governing acute care services across the Canadian province of Newfoundland and Labrador completed an anonymous questionnaire regarding their use of eight RBPs and associated P&Ps. Data were also obtained from authorities in six of the eight regions about existing relevant P&Ps. We used descriptive statistics and multivariate regression analysis to assess the relationship between key independent variables and self-reported use of RBP.

**Results:**

Use of the eight RBPs ranged from 7.8% to 88.6%, depending on the practice. Nurses ranked P&P manuals as their number one source of practice knowledge. Most respondents (84.8%) reported that the main reason they consult the P&P manual is to confirm they are practicing according to agency rules. Multivariate regression analysis identified three significant predictors of being a user versus non-user of RBP overall: awareness, awareness by regular use, and persuasion. Six significant predictors of being a consistent versus less consistent user of RBP overall were also identified: perception of P&P existence, unit, nursing experience, personal experience as a source of practice knowledge, number of existing research-based P&Ps, and lack of time as a barrier to consulting P&P manuals.

**Conclusion:**

Findings suggest that nurses use P&Ps to guide their practice. However, the mere existence of P&Ps is not sufficient to translate research into nursing practice. Individual and organizational factors related to nurses' understanding and use of P&Ps also play key roles. Thus, moving research evidence into practice will require careful interplay between the organization and the individual. P&Ps may be the interface through which this occurs.

## Background

One of the goals of a practice discipline is to strengthen the scientific foundation of clinical practice. The use of research evidence is an accepted way to achieve this goal and in turn can improve nursing care, optimize patient outcomes, and decrease costs [1,2]. Despite these potential benefits, a number of investigators have confirmed that a gap exists between research and its direct application to nursing practice. Early studies indicated that nurses were unaware of or were using research findings in their practice inconsistently [3,4]. More recent studies have shown that while both awareness and overall adoption of research-based nursing practices have increased, use remains inconsistent [5-8].

Early efforts to increase research use in nurses focused on strategies aimed at the individual nurse. Such strategies included increasing the reading activity of nurses, teaching research critique and appraisal skills, and offering a variety of educational programs targeted at the individual [9]. More recently, investigators have called for organizational approaches [9,10]. Most of the evidence in support of an organizational approach to promote research use by nurses comes from investigations of the barriers nurses perceive to using research in practice. This work consistently shows that nurses perceive organizational barriers to be the most problematic. In particular, they perceive that lack of authority, time, and support interferes with their ability to implement research findings in practice [11-14]. Further support for an organizational approach comes from recent studies examining the sources of knowledge that nurses draw upon in their daily practice. These studies consistently show that nurses rely on knowledge sources that are embedded in organizational processes such as in-service education, policy and procedure (P&P) manuals, and discussions with physicians and fellow nurses [15-17].

Revising and updating P&P manuals based on the best available research evidence has been proposed as one organizational strategy for increasing research use by nurses [5,7]. Organizational P&Ps are guideline-like documents developed by organizations to guide employees in their daily work. Limited research to date has examined nurses' understanding or their use of P&Ps. However, four studies have investigated nurses' perceptions of the existence of P&Ps related to their use of specific research-based practices (RBPs). All four studies, conducted in the United States, found that perceiving or believing that a policy existed was associated with increased use of RBPs [4-7], regardless of whether an actual policy existed [6]. However, these studies did not investigate either the direction of the relationship or whether policy perception leads to behavior change (i.e., adoption of RBPs), or whether behavior change leads to the perception that a policy exists.

In summary, we do not know the extent to which nurses are using research as a basis for quality patient care, nor do we know the role that P&Ps may play in promoting research use. While preliminary studies suggest that using P&Ps may be a promising strategy to promote research utilization, there is not sufficient evidence to make a generalization regarding the use of P&Ps to achieve this goal. Considerable resources are necessary to develop new P&Ps, revise existing manuals based on current research evidence, and disseminate these P&Ps to staff nurses. Thus, before we invest further resources in this approach, a broader understanding of the use of P&Ps by nurses and their relationship to research utilization is needed. The primary aim of this research study was to identify factors influencing the use of RBPs among staff nurses in the Canadian province of Newfoundland and Labrador with the specific aim of understanding the role of P&Ps in promoting research utilization.

### Theoretical framing

**Figure 1:**
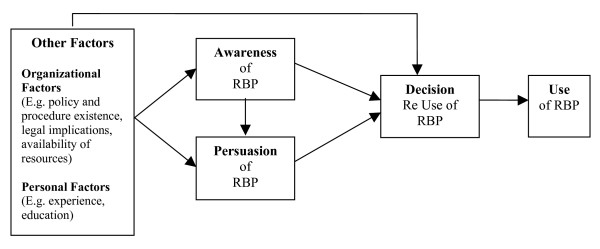
Decision-Making and Use of Research-Based Practices Model.

No theory or conceptual model currently exists to explain the role P&Ps may play in promoting research use. Therefore, using Rogers's diffusion of innovations theory [18,19] as a basis, we developed the decision-making and use of research-based practices model to guide this study (see Figure 1).

Rogers' innovation decision process theory, a component of his diffusion of innovations theory [18,19], has been used in numerous studies examining the adoption of RBPs by nurses [5-8,20-22]. This theory is based on five distinct stages which progress in a linear fashion: knowledge (or awareness), persuasion, decision, use (or implementation), and confirmation. Research over the years has confirmed that these stages do exist [5-8,20-22]. However, in recent years, diffusion researchers have come to emphasize the complex and non-linear nature of innovation adoption [23]. As depicted in our decision-making and use of research-based practices model, some stages in Rogers' innovation decision process theory may be skipped. For example, awareness may lead directly to use without persuasion occurring [6,8]. Other factors, in addition to awareness and persuasion, may also influence a nurse's decision to use a practice. These factors may include, but are not limited to, personal factors such as clinical experience and education, as well as organizational factors such as available resources, the existence of P&Ps relating to a practice, and the importance that organizations place on these P&Ps.

### Research questions

The primary research questions addressed in this paper are:

1. Do nurses use policies and procedures to guide their nursing practice?

2. To what extent have eight specific research-based practices been adopted by nurses?

3. What factors influence nurses' use of research-based practices?

## Methods

### Design

This was a cross-sectional survey study of staff nurses and agency resource nurses (ARNs), employed in acute care institutions across one eastern Canadian province-Newfoundland and Labrador. Ethical approval for the study was obtained from the Human Investigations Committee at Memorial University of Newfoundland.

### Agency resource nurse sample

#### Participants

ARNs are nurses involved in P&P development. ARNs for six of the eight healthcare regions (one ARN per region) governing acute care services across Newfoundland and Labrador participated in the study for a total ARN sample of six and response rate of 75%.

#### Data collection

ARNs completed the 35-item agency resource nurse questionnaire (ARNQ) either as a self-administered mailed questionnaire or answered the same questions in a telephone interview.

#### Instrument

We developed the ARNQ for this study based on an earlier published interview guide [24] and a critical review of the literature. The ARNQ consists of three sections. Section A, current policies and procedures, consists of a checklist of eight RBPs (which are also included in the Staff Nurse Questionnaire) and requested ARNs to indicate whether their region currently had P&Ps related to any of these eight practices. Section B, policy and procedure development, asked ARNs a variety of open and closed-ended questions related to developing and revising P&Ps. Section C, related policies and procedures, consisted of a list of the eight RBPs and requested ARNs to collect and return to the researcher anonymized copies of any P&Ps related to them.

This paper will provide an overview of the existence of research-based P&Ps related to eight specific practices. A region was considered to have adopted some or all of the eight practices if their current P&Ps on them were research-based. P&P existence was determined through an assessment of returned P&Ps where available, and through ARN statements of P&P existence where no P&Ps were returned. Accuracy of these statements was assumed because all P&Ps returned matched ARN statements with respect to policy existence. The processes used to develop and disseminate P&Ps will be presented elsewhere.

### Staff nurse sample

#### Participants

All registered nurses practicing in an adult medical, surgical, and/or critical care unit in an acute care institution and who agreed on their 2003 nurse licensure renewal to have their names released for research purposes were asked to complete a survey (n = 464). Medical, surgical, and critical care nurses were chosen to permit comparison with previous studies. A total of 248 nurses returned surveys for a response rate of 53.5%. A demographic profile of the staff nurses is listed in Table 1. According to the Association of Registered Nurses of Newfoundland and Labrador statistics, this sample is similar to the practicing nurse workforce in the province with respect to nursing experience, education level, employment status, and primary unit of employment (H. Hawkins, Personal Communication, 6 January, 2004).

**Table 1:** Demographic characteristics of participant nurses (n = 248)

**Characteristic**	**% (n)**^1^	**Characteristic**	**% (n)**^1^
**REGION**		**RESEARCH EXPERIENCE**	
Region 1	58.1% (144)	None	48.0% (119)
Region 2	4.8% (12)	As a participant	32.3% (80)
Region 3	7.3% (18)	Data Collection	10.5% (26)
Region 4	6.0% (15)	Other^3^	9.2% (23)
Region 5	8.9% (22)		
Region 6/7^2^	3.2% (8)		
Region 8	11.7% (29)		

**CLINICAL AREA**		**EDUCATION**	
Critical Care	35.9% (89)	RN Diploma	40.3% (100)
Medicine	23.4% (58)	RN Diploma + Specialty Course	22.2% (55)
Surgery	22.2% (55)	Bachelor's Degree	37.1% (92)
Med-Surg Unit	18.5% (46)	Master's Degree	0.4% (1)

**EMPLOYMENT STATUS**		**P&P DEVELOPMENT**	
Full-Time	76.6% (190)	Never	78.6% (195)
Part-Time	14.9% (37)	Currently or recently (past 12 months)	8.5% (21)
Temporary/Casual	8.5% (21)	More then 12 months ago	12.9% (32)

**YEARS IN NURSING**		**EDUCATION PROGRAM**	
Mean (SD)	11.4 (8.2)	Yes	13.7% (34)

**YEARS IN CURRENT POSITION**		No	86.3% (214)
Mean (SD)	6.6 (5.7)		

^1 ^% and n = Proportion and number of nurses with the specified characteristics

^2^region 6/7 = Regions 6 and 7 were combined to keep the identity of the individual institutions confidential

^3^Other = Study design/analysis/writing up results/presenting results

SD = standard deviation

#### Data collection

The staff nurses completed an anonymous self-administered mailed questionnaire – the staff nurse questionnaire (SNQ). All nurses identified were mailed a copy of the SNQ and a reply card to indicate whether they had completed and returned their questionnaire. Approximately one month later, a reminder letter, along with a replacement questionnaire, was sent to all nurses from whom reply cards were not received.

#### Instrument

The SNQ consisted of 96 items divided into four sections: demographic data, nursing practice, sources of knowledge, and P&P use. The content of the SNQ was based on a critical review of the literature and the study's conceptual framework, and was reviewed by an organizational P&P expert consultant. The demographic and P&P sections were newly developed for the study (see Additional File 1).

The nursing practice section is a revised version of Brett's [6] nurses practice questionnaire (NPQ). It lists eight RBPs followed by a series of seven questions. The first six questions measure the nurse's stage of adoption according to Roger's diffusion of innovations theory while the seventh question measures policy perception. The awareness stage was measured by questions one to four. An answer of 'yes' to any one of these four questions is scored as one while 'no' is scored as 0. The fifth question measures the nurse's belief in the value of the nursing practice (persuasion) and a response of 'yes' adds one point to the score. The sixth question measures implementation (use) of the practice. A response of 'use sometimes' adds one point while a response of 'use always' adds two points to the score. The range of possible scores for each practice (zero to four) is summed and averaged to give a mean total innovation adoption behavior (TIAB) score. The seventh question assesses nurses' perception of the existence of organizational P&Ps related to the eight practices. This question is scored dichotomously, as yes or no, and is not used in the calculation of the TIAB score.

Brett showed the original NPQ to be a reliable measure with a Chronbach alpha of 0.95 for the instrument and a test-retest reliability of r = 0.83 using a one-week interval [6]. Coyle and Sokop [5] replicated Brett's study and reported an alpha co-efficient of 0.91 for the instrument. Similar ranges have also been reported in subsequent studies [7,8,20,21] indicating the NPQ is a reliable instrument for measuring adoption of specific RBPs. In our study we obtained an alpha coefficient of 0.82 for the revised NPQ. Content validity of the NPQ has always been assumed, as the practices used have been derived from published research reports using specific criteria. No formal validity testing has been carried out.

The sources of knowledge section was a direct replication of Estabrooks' [15] sources of knowledge questionnaire, which is an adaptation of Baessler et al. instrument [25]. It asks nurses to rate the frequency with which they use 16 different sources of knowledge in their practice. Each source is scored using a five-point scale from never use (one) to always use (five). The frequency with which nurses use the various sources of knowledge is reflected in the mean scores obtained for each source. In this study, an alpha coefficient of 0.78 was obtained for the scale. No formal reliability or validity testing has been reported on this scale in previous studies.

Prior to being used in the main study, the SNQ was tested for feasibility with a small group of staff nurses (n = 12). The questionnaire was found to be readable and brief, taking approximately 15 minutes to complete. Based on the feasibility test no revisions to the SNQ were necessary.

### Selection of RBPs

To identify RBPs, we conducted a search of the Cochrane database of systematic reviews and the database of abstracts of reviews of effectiveness, the CINAHL and MEDLINE electronic databases, and the Internet. Practices were selected if they: 1) could be implemented by individual nurses, 2) were general and applicable to the practice of adult medical/surgical nurses, and 3) were supported by a systematic or synthesized review, meta-analysis, or clinical practice guideline. Only eight practices were located that met all three criteria. For a list of the eight practices selected and their supporting references see Additional File 2.

### Data analysis

Data were analyzed using the SPSS [v. 13.0] and Stata (Stata 9) statistical programs. Descriptive statistics were used to summarize the data. For dichotomous variables, cross-tabulations and chi-square tests were conducted. Multivariate analysis using stepwise logistic regression with cluster correction for region was performed to determine which factors were significant predictors of being a user/nonuser and consistent/less consistent user of RBP. All open-ended questions were analyzed using thematic content analysis, as outlined in Morse and Field [26]. We first generated word frequency lists from the open-ended question data, from which common themes (categories) were identified. Category counts were then tabulated for each open ended question and these counts were used to summarize the data.

## Results

The study results are reported under the three research questions addressed in this paper.

### Do nurses use policies and procedures to guide their nursing practice?

#### Sources of knowledge

Using the sources of knowledge questionnaire [15], nurses were asked to indicate the extent to which they use 16 sources of knowledge, including P&P manuals, in their practice. Most nurses (81.9%) reported using the knowledge they obtain from P&P manuals frequently or all the time in their daily practice. Other sources frequently/always used by a majority of nurses included knowledge obtained from: personal experience (81.1%), nursing school (75.8%), each individual patient (74.6%), and in-services/conferences (69.3%) (See Table 2).

**Table 2:** Frequency of use of the 16 sources of knowledge (n = 248)

***Knowledge Obtained From:***	***Mean Score (SD)***^1^	***Frequency of Use % (n)^2^***		
		
		Never/Seldom	Sometimes	Frequently/Always
Policy and Procedure Manuals	4.19 (.85)	5.2% (13)	12.9% (32)	81.9% (203)
Personal Experience	4.00 (.73)	2.8% (7)	16.1% (40)	81.1% (201)
Nursing School	4.00 (.76)	1.6% (4)	22.6% (56)	75.8% (188)
Individual Patient	3.95 (.80)	3.2% (8)	22.2% (55)	74.6% (185)
Physician Discussions	3.86 (.73)	3.2% (8)	28.2% (70)	68.6% (170)
In-services/Conferences	3.84 (.84)	8.1% (20)	22.6% (56)	69.3% (172)
Doctor's Orders	3.74 (.82)	6.5% (16)	36.7% (91)	56.8% (141)
Fellow Nurses	3.61 (.66)	2.8% (7)	38.7% (96)	58.5% (145)
Worked for Years	3.60 (.81)	11.3% (28)	37.1% (92)	51.6% (128)
Text Books	3.55 (.83)	9.3% (23)	32.7% (81)	58.0% (144)
Intuition	3.40 (.90)	12.1% (30)	40.3% (100)	47.6% (118)
Way Always Done It	3.18 (.84)	21.8% (54)	45.6% (113)	32.6% (81)
Nursing Journals	3.07 (.89)	28.2% (70)	43.6% (108)	28.2% (70)
Medical Journals	2.92 (.93)	36.7% (91)	41.1% (102)	22.2% (55)
Nursing Research Journals	2.75 (.96)	41.1% (102)	37.1% (92)	21.8% (54)
Media	2.53 (.88)	58.1% (144)	31.9% (79)	10.0% (25)

^1 ^Mean (SD) = mean score (average based on the following scale: never-1, seldom-2, sometimes-3, frequently-4, and always-5) and standard deviation associated with each of the identified sources

^2 ^Frequency of Use % (n) = proportion and number of all 248 nurses who reported never/seldom, sometimes, and frequently/always using the selected sources of knowledge

#### Use of policies and procedures

All nurses reported they consult their institution's P&P manual(s) at least sometimes, and almost a third (32.9%) always did. Most (78.0%) reported they always adopt their institution's P&Ps, and that they are among the first on their unit to do so (74.6%). Only 6.9% indicated they would consider incorporating research findings into practice that were not supported by a current P&P.

#### Understanding of policies and procedures

The majority (80.4%) agreed that the purpose of P&P manuals was to guide nursing practice, while others felt it related to standards of care (23.7%), consistency of care (22.9%), and legalities (17.1%) such as protection for the hospital or nurse. To further assess the nurses' understanding of P&Ps, they were asked, in an open-ended question, if they could distinguish between a policy and a procedure. Just over a third (38.8%) could correctly differentiate between a policy and a procedure. Less than a quarter (20.0%) felt there was no difference, while 41.2% were incorrect in their distinction. Many of the nurses that were incorrect could neither define a policy nor a procedure (48.8%), while equal proportions of the nurses who were incorrect could correctly define one term but not the other.

The nurses reported that they consult P&P manuals for the following reasons: confirm institutional policy (84.8%), new and unfamiliar tasks (41.8%), to ensure a task is within their scope of practice (8.6%), to settle disputes regarding the correct way to perform a task (5.7%), and to teach students and orientate new staff (4.5%). Reasons reported for not consulting P&P manual(s) more frequently included: familiar and routine tasks (51.6%), lack of time (43.0%), difficulties with the manual(s) (21.0%) such as missing pages, and, availability of co-workers who know the P&P in question (15.1%).

Staff nurses were also asked about the legal implications of not following their agency's P&Ps. Most nurses reported not knowing the legal implications (33.1%) or perceiving criminal charges (40.9%) and/or discipline from their employer (37.6%) as possible consequences. A small percentage (10.3%) also reported discipline from their nurse licensing body in the form of loss or suspension of their nursing license.

### To what extent have eight specific research-based practices been adopted by nurses?

The percentage of nurses reporting awareness of the RBPs ranged from 12.9% to 89.1%, persuasion from 23.0% to 86.7%, and any use from 7.8% to 88.6%. Any use refers to sometimes or always use of the practice. Those reporting sometimes use ranged from 3.7% to 59.6% and always use from 2.0% to 74.8%. The mean total innovation adoption behavior (TIAB) score for each practice ranged from 0.47 (unaware stage of adoption) to 3.38 (use sometimes stage of adoption) using Brett's [6] classification system (see Table 3).

**Table 3:** Adoption of the 8 research-based practices (n = 248)

**Practice**	**Aware (%^1^)**	**Persuaded (%^1^)**	**Use (%^2^)**			**Mean TIAB (SD)^3^**	**No. Regions with P&Ps (n = 6)**
					
			Never	Sometimes	Always		
Flushing PIVs	89.1%	86.7%	13.4%	11.8%	74.8%	3.38 (1.24)	4
Urinary Catheter Care	87.5%	86.7%	11.4%	17.5%	71.1%	3.34 (1.22)	4
Compression Stockings	89.1%	83.5%	18.3%	59.6%	22.1%	2.76 (1.10)	2
Hyperoygenating	78.6%	79.4%	36.2%	25.1%	38.7%	2.61 (1.47)	4
CHG for IV Insertion	67.3%	65.3%	38.3%	16.5%	45.2%	2.40 (1.73)	2
Post-op Opiod Admin	66.1%	62.5%	36.4%	46.6%	17.0%	2.10 (1.44)	1
NG Tube Placement	34.7%	23.0%	91.5%	6.5%	2.0%	0.68 (1.03)	2
Closed Enteral Feeding	12.9%	23.8%	92.2%	3.7%	4.1%	0.47 (1.00)	0

^1^% = Percentage of nurses who were aware and persuaded of the practice

^2^% = Percentage of nurses who were never, sometimes, and always used the practice, of those who did not rate the practice as not applicable to their clinical unit

^3^Mean TIAB (SD) = mean TIAB score and standard deviation associated with each of the identified practices. The mean TIAB score was calculated as the average of the sum of awareness (0 – 1) + persuasion (0 – 1) + use (0 – 2). (Possible range: 0 – 4).

Stage of adoption was classified according to the following scale: 0 – 0.49 (unaware), 0.5 – 1.49 (aware), 1.5 – 2.49 (persuasion), 2.5 – 3.49 (use sometimes), and 3.5 – 4.0 (use always)

The three RBPs that nurses were the most aware of were the same practices they were the most persuaded of and used the most: flushing peripheral locks, urinary catheter care, and using graduated compression stockings. More than 80% of nurses indicated being aware of, persuaded of, and using (at least sometimes) these three practices. Using Brett's [6] classification system of mean TIAB scores, these three practices were classified as being in the use sometimes stage of adoption. The practice of hyperoxygenating patients prior to suctioning was also in the use sometimes stage of adoption. However, while similar in awareness and persuasion to the other three practices, fewer nurses reported using this practice.

The practices of using chlorhexidine gluconate prior to IV insertion and postoperative analgesic administration were moderately-known and used by this sample of nurses as follows. Between 61.7% and 67.3% of nurses reported being aware of, persuaded of, and using these two practices at least sometimes. Both practices were categorized as being in the persuasion stage of adoption.

The remaining two practices, Nasogastric (NG) tube placement and closed enteral feeding, were the least-known, persuaded of, and used practices. Only between 12.9% and 34.7% of nurses reported being aware of and/or persuaded of these two practices. Additionally, less than 10% of nurses reported any use of these practices. The practice of NG tube placement was categorized as being in the aware only stage of adoption while using closed systems for enteral feeding was in the unaware stage of adoption.

### Policy perception, policy existence, and adoption

Examination of ARN statements made regarding existence of P&Ps on the eight RBPs showed one of the six participating regions did not have research-based P&Ps on any of the practices. Of the remaining five regions, one region had research-based P&Ps for three of the practices and four had research-based P&Ps for four of the practices. Using the Fishers exact test statistic we examined the relationship between nurses' use of each practice and: 1) the existence of P&Ps on each practice, and 2) nurses' perception of P&P existence on each practice. P&P existence was only significantly associated with the use of one practice: chlorhexidine gluconate prior to IV insertion (p < 0.05). However, staff nurse perception of P&P existence was significantly associated with using all eight practices (p < 0.05). Significantly higher proportions of nurses who reported any use compared to no use of the eight practices perceived P&Ps to exist on them. Furthermore, for six of the eight practices significantly higher proportions of nurses who reported always using compared to sometimes using them perceived P&Ps to exist on them. The only exceptions were the practices of NG tube placement and closed enteral feeding systems.

### What factors influence nurses' use of research-based practices?

To determine which factors influence the use of RBPs overall, nurses were classified as users/nonusers and as consistent/less consistent users of RBP. For this classification, data from six of the eight practices were pooled. Two practices, verifying NG tube placement and using closed enteral feeding systems, were omitted from the pooled dataset since less than 10% of the nurses surveyed reported any use of them. Using the pooled dataset, nurses who reported any use for at least four of the six practices (or three of the five practices if one practice was rated as not applicable) were classified as users of RBP. All remaining nurses were classified as nonusers. Nurses who rated more then one practice as not applicable were excluded from the analysis. This resulted in six nurses being excluded for a final sample of 242. Of these 242, 193 (79.8%) were classified as users, and 49 (20.8%) as nonusers. This process was repeated to classify the 193 users into consistent (or always) and less consistent (or sometimes) users. This resulted in 61 (31.6%) consistent users and 132 (68.4%) less consistent users. This process was also used to classify nurses as being: aware, aware by regular use, persuaded, policy perceivers, and correct policy perceivers. A stepwise logistic regression modeling process, adjusting for the six clusters in region, was then carried out for users versus nonusers and consistent versus less consistent users of RBP.

### User versus nonuser

All factors shown to have statistically significant associations in bivariate analysis with being a user versus nonuser of RBP, along with the number of existing research-based P&Ps related to the eight practices (to control for policy existence), were entered into the initial model (see Table 4). The regression equation for the final model was: In odds of being an user/nonuser = B_0 _+ B_1_(Awareness) + B_2_(Aware_reg_use) + B_3 _(Persuasion) + Error, in which 'Awareness' indicates overall awareness of the practices, 'Aware_reg_use' indicates being aware by seeing others carry out the practice, and 'Persuasion' indicates being persuaded the practices are the most appropriate ones. The final model suggests users of RBP were more likely to be: aware overall, aware by regular use, and persuaded of the appropriateness of a practice compared to nonusers (see Table 4). The R^2 ^for the final model, 0.5415, indicated that these three variables together accounted for 54.15% of the variance of being a user of RBP.

**Table 4:** Regression analysis

**Variable**	**B_0 _Coefficient (95% CI)**	**Odds Ratio (95% CI)**	**P value**
**User vs. Nonuser of RBP**			

**Awareness**	**2.52 (0.98, 4.06)**	**12.40 (2.65, 57.89)**	**0.001**
**Awareness by regular use**	**3.49 (2.47, 4.50)**	**32.78 (11.87, 90.45)**	**0.001**
Education Level	-1.12 (-2.34, 0.11)	0.33 (0.09, 1.11)	0.074
**Persuasion**	**2.11 (0.40, 3.83)**	**8.28 (1.49, 46.04)**	**0.016**
Policy Perception	-0.21 (-1.11, 0.69)	0.81 (0.32, 2.00)	0.651
Correct policy perception	0.35 (-0.81, 1.52)	1.42 (0.44, 4.59)	0.555
P&P manual- source of knowledge	0.25 (-0.60, 1.10)	1.28 (0.54, 3.01)	0.562
In-services – source of knowledge	0.34 (-0.47, 1.15)	1.40 (0.62, 3.16)	0.412
Unaware of legal implications	-0.88 (-1.94, 0.18)	0.41 (0.14, 1.19)	0.104
# Research-Based P&Ps	0.14 (-0.21, 0.48)	1.14 (0.81, 1.60)	0.437

**Consistent User vs. Less Consistent User of RBP**			

**Lack of time**	**-0.69 (-1.17,-0.21)**	**0.50 (0.30, 0.80)**	**0.005**
Awareness	-1.99 (-5.46, 1.49)	0.13 (0.01, 4.42)	0.262
Awareness by regular use	3.41 (-0.29, 7.12)	30.39 (0.74, 123.72)	0.071
Education Level	0.15 (-0.41, 0.70)	1.15 (0.66, 2.00)	0.605
Persuasion	-1.72 (-5.21, 1.77)	0.17 (0.01, 5.88)	0.334
**Policy Perception**	**0.58 (0.09, 1.07)**	**1.78 (1.09, 2.91)**	**0.020**
**Unit **(critical care-reference group)	**-0.42 (-0.72,-0.12)**	**0.65 (0.48, 0.88)**	**0.006**
**Experience in nursing**	**0.07 (0.02, 0.12)**	**1.07 (1.01, 1.12)**	**0.009**
Employment status	0.09 (-0.25, 0.42)	1.08 (0.78, 1.56)	0.611
**Experience-source of knowledge**	**0.55 (0.24, 0.87)**	**1.74 (1.26, 2.39)**	**0.001**
P&P manual-source of knowledge	0.36 (-0.07, 0.78)	1.42 (0.93, 2.18)	0.101
In-services-source of knowledge	-0.59 (-1.28, 0.09)	0.55 (0.27, 1.10)	0.092
Purpose of P&Ps – guide practice	-0.99 (-2.59, 0.61)	0.37 (0.07, 1.83)	0.225
**# Research-Based P&Ps**	**0.24 (0.05, 0.42)**	**1.26 (1.05, 1.52)**	**0.012**

### Consistent versus less consistent users

All factors shown to have statistically significant associations with being a consistent versus less consistent use of RBP in the bivariate analysis (see Table 4) along with the number of existing research-based P&Ps related to the eight practices (to control for policy existence) were entered into the initial model. The regression equation for the final model was: in odds of being a consistent user/less consistent user = B_0 _+ B_1_(Policy_perc) + B_2_(Unit) + B_3 _(Experience) + B_4 _(Per_exp) + B_5 _(No_ policies) + B_6 _(Lack_time) + Error, in which 'Policy_perc' indicates perceiving P&Ps to exist, 'Unit' indicates the nurse's primary area of employment, 'Experience' refers to the length of time (in years) one has practiced as a nurse, 'Per_exp' indicates personal experience as a source of knowledge, 'No_policies' indicates the number of existing research-based P&Ps related to the eight practices surveyed, and 'Lack_time' indicates perceiving lack of time as a barrier to using P&P manuals. The final model suggests that consistent users of RBP were more likely to perceive P&Ps to exist, have higher levels of nursing experience, rely on personal experience as a source of knowledge, and work in critical care units (compared to medical or surgical units). Consistent users were also less likely to view lack of time as a barrier to consulting the P&P manual (odds ratio < 1) (see Table 4), The R^2 ^for the final model, 0.3190, indicated that together these six variables accounted for 31.90% of the variance of being a consistent user of RBP.

## Discussion

### Adoption of research-based practices

The adoption scores obtained are comparable to those found in earlier studies by Brett [6] and Coyle and Sokop [5], but appear lower then those reported in more recent studies by Rutledge et al. [21] and Rodgers [8] (see Additional File 3). One possible explanation for this is that Rutledge and colleagues only included nurses who had reported being aware of their practices in their calculation of adoption scores. When we only included aware nurses, an overall adoption score (3.39 – use sometimes stage) similar to that reported by Rutledge et al. (3.33 – use sometimes stage) was obtained. Rodgers, on the other hand, may have found higher adoption scores due to the types of practices selected. We only investigated practices that could be implemented directly by staff nurses (i.e., instrumental research use), whereas Rodgers also included three practices that could be indirectly utilized by nurses (i.e., conceptual research use). Several studies have confirmed that conceptual research use tends to be the most frequent form of research use [27-29] and consequently, lends itself to over-reporting by respondents [29]. The three indirect practices selected by Rodgers obtained high mean adoption scores, correlating with Brett's use always stage of adoption. Using data published in Rogers' study, a mean adoption score in the persuasion stage which is comparable to that found in our study and others would be obtained if these three practices were omitted. Thus, our results are actually similar to recent previous studies. But what is of concern is that that there has been *no *improvement in the uptake of RBPs by nurses.

Using Brett's [6] classification system, the mean adoption score for all eight practices combined placed nurses in this study in the persuasion stage of adoption, overall. In other words, they were aware of and believed in the value of the practices but were not using them to make clinical decisions. However, given that over half of the nurses in this study reported using a majority of the practices at least sometimes (see Table 3), Brett's categories of aware only, persuasion, use sometimes, and use always do not appear to accurately reflect the extent of adoption in this study. Hence, while Brett's classification system was used in this study to allow comparisons to be made with previous studies, presenting adoption scores without attaching fixed labels to them may be a more appropriate way of discussing adoption behavior in future studies.

There was variability in the nurses' adoption of the eight practices. Some practices appeared to be well adopted while others were poorly adopted (see Table 3) despite being easy to implement and having little cost. Several factors, personal and organizational, as depicted in our decision-making and use of research-based practices model, may account for this variability. For example, for the practice of using graduated compression stockings, physician orders may play a role. For other practices, nursing beliefs may play a role. For instance, despite strong evidence to support regular opioid administration post-operatively [30] some nurses may remain reluctant to administer these medications even when ordered (e.g., q4h PRN) due to fear of dependence or the development of serious side effects. Patient preferences may also play a role in deciding to implement these practices. For instance, a patient may wish not to be medicated regularly or may refuse to wear compression stockings.

Availability of resources and cost may also account for some of the noted variability. For instance, heparin and antimicrobial solutions are more expensive then normal saline and soap and water, and therefore would be less available in some institutions. This may account for why the practices of flushing peripheral locks with normal saline and performing urinary catheter care with soap and water have been widely adopted. Similarly, a lack of pH paper in some institutions may account for why many nurses have not adopted the practice of verifying NG tube placement by checking pH levels. Thus, innovation adoption is not as straightforward as a reading of Rogers [18,19] could suggest. There are multiple factors, personal and organizational, that may account for why some practices are adopted more often then others.

### Policies and procedures and adoption of research-based practice

#### Value of policies and procedures to nurses

Our findings suggest that nurses use P&Ps to guide their practice. P&P manuals were the number one source of knowledge in the current study. P&Ps were also highly relied on by nurses in past studies; they were the fifth leading source of knowledge in studies conducted by Estabrooks [15] and Palfreyman and colleagues [17], sixth in a study conducted by Gerrish and Clayton [13], and eighth in Estabrooks' [16] most recent study on sources of knowledge. Knowledge obtained from nursing school and experiential knowledge acquired through interactions with patients were also used frequently, while knowledge obtained from literature, namely professional journals, and the media were not. This is also consistent with past studies [13,15-17,25].

Some interesting issues arise from these results. First, P&Ps were reported as the top source of practice knowledge in our study. Therefore, they hold potential to keep nurses informed of advances in research. Also important is the ongoing role that basic nursing education appears to play as a source of practice knowledge. Nursing school was the third most frequently used source of knowledge for this sample of nurses, even though they completed their basic nursing education program an average of 12.5 years ago. While much of the knowledge they obtained in their basic nursing program remains valid, much of what was learned, especially around clinical techniques, has changed. For example, 15 years ago schools of nursing taught their students that the proper method to verify NG tube placement was to inject air into the tube and listen for air entry over the epigastric region, or to place the free end of tube into a glass of water and check for bubbling. Not only are these methods now known to be ineffective, but they can also be potentially harmful to the patient if the tube is misplaced and consequently used [31-33].

Another important finding is that nurses rely heavily on experiential knowledge rather than on knowledge from scientific and professional literature. One reason for this may relate to accessibility. Many nurses in this study, especially those in rural settings, work in units where availability of computers, the internet, and research journals is limited. Similarly, accessibility may also explain the increased tendency seen in this study for nurses to rely on P&P manuals. The culture of nursing work often means nurses are confined to their unit and do not have time to leave their patients to access computers or search the professional literature. Unlike research journals, P&P manuals are located on all nursing units and are thus easily accessible to all nurses. Furthermore, less time is needed to look up a P&P in a manual then is needed to perform a search for available research on a practice and then locate, read, and critically appraise that research.

#### Policy perception and existence

Consistent with previous studies [5-7] we found staff nurse perception that a P&P existed was a significant predictor of using RBPs. In addition to policy perception, Brett [6] also investigated policy existence and found that it was not related to individual nurse adoption of specific practices. This finding is consistent with our study, which found that the existence of practice policies was significantly associated with the use of only one of the eight practices examined. However, we went a step further and examined whether the number of existing research-based P&Ps in an institution influenced use of RBP overall. Multivariate analysis showed that working in a region with more research-based P&Ps was a significant predictor of consistent use of RBP. Thus, contrary to Brett's finding, we conclude that policy existence may be important to the adoption of RBPs by nurses. However, we suggest it is the number of existing research-based P&Ps that is important, not the mere existence of specific P&Ps. However, further research in this area is needed before any firm conclusions can be drawn.

#### Understanding of policies and procedures

All nurses should have a basic understanding of what P&Ps are, and when and how to use them. Fear of the legal ramifications of not following P&Ps may be one explanation why some nurses accept and follow P&Ps even when they are not based on best evidence. In this study, 33.3% of nurses reported being unaware of the legal implications of not following their institution's P&Ps, while the remainder perceived devastating consequences. If we are to use P&Ps to promote research utilization and improve the quality of care that nurses deliver, nurses and managers need to be aware of the implications, legal and professional, of not only not following organizational P&Ps, but also of continuing to follow P&Ps when they know they are not based on the best available evidence. However, even more importantly, service organizations and professional associations and unions must work to decrease the punitive connotation to P&P non-compliance. Continuing education for managers and administrators, educators, and clinicians should incorporate relevant content on P&Ps.

### Future research

While this study, and others, sheds some light on the factors influencing nurses' use of RBP, further research is needed to expand on this knowledge. Specifically, this was the first study to go beyond P&P perception and existence to investigate P&P understanding in nurses and therefore should be replicated, preferably with a larger probability sample, to see if findings remain consistent across future studies. In addition, one specific finding which warrants further research is the variable 'awareness by regular use'. This variable was shown to be a significant predictor of being a user of RBP overall. However, it is not clear from the data collected what exactly the true role of regular use is. Awareness by regular use was not defined for the study participants but left up to them to interpret. Therefore, we recommend that future studies on research utilization investigate this variable further.

### Study limitations

There are a number of limitations in this study. First, the nurses were alerted to the importance of P&Ps in an explanatory letter that accompanied the mailing of their questionnaire. This may have resulted in a higher reported rate of reliance on P&Ps than actually existed. Second, there has been no formal validity testing done of the nurses practice questionnaire (Section B of our staff nurse questionnaire). Third, while this was a multi-center study, the relatively small sample size of 248 nurses may have been inadequate to detect real differences for some of the variables. Fourth, the variable 'awareness by regular use' was shown to be a significant predictor of being a user of RBP. However, we could not determine what the role of regular use was or what it means from our data. Fifth, ARNs could not be recruited for two of the eight healthcare regions. As a result, findings from this study cannot be generalized to all regions in the province. Finally, although the sample was similar to the target population with respect to experience, education, unit, and employment status, the available population represented only a third of the target population, and therefore generalizability beyond the study sample is limited.

## Conclusion

Consideration of the findings of this study within the context of our decision-making and use of research-based practices model and previous studies on research utilization make it possible to gain a better understanding of the factors that influence nurses' use of RBPs, and the role that P&Ps may play in promoting research utilization in nurses. Our findings suggest nurses use P&Ps to guide their practice. However, the mere existence of research-based P&Ps is not enough to increase research utilization in nurses. Individual and organizational factors related to nurses' understanding and use of P&Ps also play key roles. Thus, we conclude that moving research evidence into practice is neither the sole responsibility of the organization nor the individual clinician. Rather, it will require careful interplay between the organization and the individual. P&Ps could be the interface through which this occurs.

## Competing interests

The author(s) declare that they have no competing interests.

## Authors' contributions

JES was responsible for the study conception and design, collecting, entering, and analyzing all data, and drafting the manuscript. JES, DM, and SML contributed to the design of the study's conceptual framework and questionnaires. All authors commented and approved the final manuscript. DM and SML supervised the study.

## Supplementary Material

Additional file 1 Newly Developed SNQ Content. This file contains sections of the Staff Nurse Questionnaire that were newly developed for this study. Click here for file

Additional file 2 Level and Source of Evidence for the 8 Selected Research-Based Practices. This file contains lists the eight research-based practices investigated in this study, and their source and level of evidence. Click here for file

Additional file 3 Adoption of Specific Research-Based Practices in Comparative Studies. This file contains a comparison of the adoption of specific research-based practices across comparable studies. Click here for file
