# Supplementary material for: Exploring the role of organizational policies and procedures in promoting research utilization in registered nurses
Source: Implement Sci. 2007 Jun 5;2:17. doi: 10.1186/1748-5908-2-17 (PMC1904235; doi:10.1186/1748-5908-2-17)
Supplement: Additional file 1 — Newly Developed SNQ Content. This file contains sections of the Staff Nurse Questionnaire that were newly developed for this study. [file 1748-5908-2-17-S1.doc]

Additional File 1. Newly Developed SNQ Content

**SNQ – Section A**

Please **ANSWER EVERY QUESTION.** Circle your response where appropriate.

1. My **highest level** of education in **nursing** is: (circle only one, i.e. highest level)
2. RN Diploma
3. RN Diploma plus Specialty Course
4. Baccalaureate Degree
5. Masters Degree or higher
6. Primary area of employment:
7. Critical Care (ICU/CCU/Dialysis)
8. Medicine
9. Surgery
10. Combination medical/ surgical unit
11. Total number of years in current position: _________________
12. Total number of years experience in nursing: _________________
13. I am employed with the:

1. Health Care Corporation of St. John’s

2. Avalon Health Care Institutions Board

3. Peninsulas Health Care Corporation

4. Central East Health Care Institutions Board

5. Central West Health Corporation

6. Grenfell Regional Health Services or Health Labrador Corporation

7. Western Health Care Corporation

1. Nature of employment:
2. Full time
3. Part time
4. Temporary
5. Casual
6. Unemployed
7. I completed my basic nursing education ________years ago
8. Are you currently enrolled in a nursing education program:

1. Yes

2. No

1. I have been involved in policy and procedure development: (e.g. policy & procedure committee)

1. Never

2. Currently or recently (past 12 months)

3. More than 12 months ago

1. My experience with nursing research has been: (circle all that apply)

1. None

2. As a participant

3. Helped with data collection

4. Study design and data analysis

5. Writing up results

6. Presenting results

**SNQ – Section D**

This section asks questions regarding the policies and procedures often found in hospital manuals. Please answer all questions. If extra space is needed you can use the reverse side of the questionnaire.

1. What do you feel is the purpose of hospital policy and procedure manuals?
2. Do you feel there is a difference between a policy and a procedure? If so, what is the difference?
3. How often do you consult your healthcare institution’s policy and procedure manual?
   1. Never (proceed to question #5)
   2. Sometimes
   3. Frequently
4. For what reasons do you consult the policy and procedure manual?
5. Why do you not consult the policy and procedure manual more frequently?
6. Are there any policies or procedures concerning nursing practice in your institution that you question?
   1. Yes
   2. No
7. If you found a hospital policy or procedure that you knew or felt was not based on the most current evidence what would you do?
8. Do you know what the legal implications are of not following your institution’s policies and procedures? If so, what are they?
9. What other factors do you take into consideration when deciding to implement a policy or procedure (e.g., research, patient preferences, your clinical expertise, and the resources available to you)?
10. Would you incorporate research findings into your practice if they were not supported by your institution’s policies and procedures?
    1. No
    2. Maybe (Sometimes)
    3. Yes

Comments:

1. How do you keep informed on new/updated policies and procedures? (e.g., in-services, email, staff meetings, looking through the manual).
2. When there is a new or revised policy or procedure, do you adopt it?
   1. Never
   2. Sometimes
   3. Always
3. When there is a new or revised policy or procedure:
   1. Are you among the first in your unit to adopt it? Yes_____ No_____
   2. Do you adopt it only after others on your unit have? Yes_____ No_____
4. How much flexibility do you feel your institution’s policies and procedures allow you in making clinical decisions?

Any additional comments can be written in the space below:
