# Supplementary material for: Exploring the role of organizational policies and procedures in promoting research utilization in registered nurses
Source: Implement Sci. 2007 Jun 5;2:17. doi: 10.1186/1748-5908-2-17 (PMC1904235; doi:10.1186/1748-5908-2-17)
Supplement: Additional file 2 — Level and Source of Evidence for the 8 Selected Research-Based Practices. This file contains lists the eight research-based practices investigated in this study, and their source and level of evidence. [file 1748-5908-2-17-S2.doc]

### *Additional File 2. Level and Source of Evidence for the Eight Selected Research-Based Practices*

| **Practice** | **Type of Study** | **Source of Evidence** |
| --- | --- | --- |
| 1. Hyperoxygenating patients who have an artificial airway prior to suctioning. | Systematic Review | Thompson, L. (2000). Suctioning adults with an artificial airway: A systematic review (The Database of abstracts of Reviews of effectiveness [DARE]). In the Cochrane Library, Issue 3, 2003. Oxford: Update Software |
| 1. Using Chlorhexidine gluconate for catheter site care reduces catheter related bloodstream infections and catheter colonization more then povidone-iodine. | Meta- Analysis | Chaiyakunapruk, N., Veenstra, D.L., Lipsky, B.A., & Saint, S. (2002). Chlorhexidine compared with povidone-iodine solution for vascular catheter-site care: A meta analysis. *Ann Intern Med,* 136, 792-801**.** |
| 1. Using graduated compression stockings, alone or in conjunction with other prophylaxis modalities, to prevent deep vein thrombosis (DVT). | Systematic Review | Amarigiri, S.V. & Less, T.A. (2000). Elastic compression stockings for prevention of deep vein thrombosis (Cochrane Review). In: The Cochrane Database of Systematic Reviews, Issue 3, 2003. Oxford: Update Software. |
| 1. Pain that is expected as a result of surgery should be anticipated and analgesics administered on a regular schedule (for the first 48 hours) to prevent the person from having to experience pain. | Clinical Practice Guideline | RNAO (2002). Nursing best practice guideline: Assessment and management of pain. Available online: [www.rnao.org/bestpractices/PDF/BPG_Assessment_of_Pain.pdf](http://www.rnao.org/bestpractices/PDF/BPG_Assessment_of_Pain.pdf)**.** |
| 1. Checking nasogatric tube placement by testing the pH and/ or bilirubin levels of tube’s aspirate are a cost-effective alternative to traditional radiograph and lessen the patient’s exposure to radiation. | Synthesized Review | Monash University, Centre for Clinical Effectiveness (2000). What is the optimum method for ensuring correct placement of nasogastric tubes? Southern Health Service/Monash Institute of Public health. Available online: http://www.crd.york.ac.uk/CRDWeb/ShowRecord.asp?View=Full&ID=32003000669 |
| 1. Urinary catheter care for patients with indwelling urinary catheters should include cleaning the meatus daily with soap and water. There is no reduction in bacteriuria when using antiseptic/ antimicrobial agents over routine bathing or showering with soap and water. | Clinical Practice Guideline | Pratt, R.J., Pellowe, C., Loveday, H.P., Robinson, N., Smith, G.W. and the guideline development team: Barrett, S., Davey, P., Harper, P., Loveday, C., McDougall, C., Mulhall, A., Privett, S., Smales, C., Taylor, l., Weller, B., & Wilcox, M. (2001). The epic project: Developing national evidence-based guidelines for preventing healthcare associated infections. Phase 1: Guidelines for preventing hospital-acquired infections, *Journal of Hospital Infection*, 47, S1-S82 |
| 1. Closed enteral feeding systems (i.e., sterile prefilled ready to use feeds that do not expose the feed to air during assembly) have lower contamination rates then open enteral feeding systems. | Clinical Practice Guideline | EPIC (2003). Guideline: Prevention of healthcare-associated infections in primary and community care. Available online**:** [http://www.epic.tvu.au.uk/PDFFiles/epic%202a/Section%204%20Enteral%20](http://www.epic.tvu.au.uk/PDFFiles/epic 2a/Section 4 Enteral ) Feeding%20June%202003.PDF |
| 1. Routinely flushing peripheral venous locks with normal saline solution is as effective as flushing with heparin unless they are being used for obtaining blood specimens, in which case a diluted heparin flush should be used. | Systematic Review | Randolph, A.G., Cook, D.J., Gonzales, C.A. & Andrew, M.A. (1998). Benefit of heparin in peripheral venous and arterial catheters: Systematic review and meta-analysis of randomized controlled trials. *BMJ*, 316, 969-975. |
