# Supplementary material for: Exploring the role of organizational policies and procedures in promoting research utilization in registered nurses
Source: Implement Sci. 2007 Jun 5;2:17. doi: 10.1186/1748-5908-2-17 (PMC1904235; doi:10.1186/1748-5908-2-17)
Supplement: Additional file 3 — Adoption of Specific Research-Based Practices in Comparative Studies. This file contains a comparison of the adoption of specific research-based practices across comparable studies. [file 1748-5908-2-17-S3.doc]

Additional File 3. Adoption of Specific Research-Based Practices in Comparative Studies

| ***Author*** | ***Number of Practices adopted by 50% or more of nurses:*** *(a/b)^1^* | | | | ***Mean TIAB Score (SD) and Stage of Adoption^2^*** |
| --- | --- | --- | --- | --- | --- |
|  | Aware | Persuaded | Used Sometimes | Used Always |  |
| Brett (1987) | 10/14 | 7/14 | 10/14 | 2/14 | 2.17 (SD = NR*) **Persuasion** |
| Coyle & Sokop (1990) | 9/14 | 8/14 | 8/14 | 0/14 | 1.96(SD = NR*) **Persuasion** |
| Michel & Sneed (1995) | - | - | - | - | 2.21(SD = NR*) **Persuasion** |
| Varcoe & Hilton (1995) | - | - | 9/10 | - | 2.15(SD = 0.36) **Persuasion** |
| Berggren (1996) | 10/14 | 8/14 | 4/14 | 3/14 | 2.06(SD = NR*) **Persuasion** |
| Rutledge et al. (1996) | 8/8 | 8/8** | 8/8** | 3/8** | 3.33^**^(SD = NR*)  **Use Sometimes** |
| Rodgers (2000) | 13/14 | 13/14 | 11/14 | 6/14 | 2.65(SD = 0.57)  **Use Sometimes** |
| **Current Study** |  |  |  |  | **2.21(SD = 0.53 )**  **Persuasion** |

^1^ (A/B) A= number of practices that 50% or more of the nurses were aware of, persuaded of, used sometimes, and used always. B=total number of practices surveyed

^2^ Mean TIAB Score (SD) and Stage of Adoption = mean total innovation adoption score, standard deviation, and stage of adoption all practices combined. TIAB score was calculated as the average of the sum of awareness (0 - 1) + persuasion (0 - 1) + use (0 - 2). (Possible range: 0 - 4). Stage of adoption classified according to the following scale: 0 – 0.49 (unaware), 0.5 – 1.49 (aware), 1.5 – 2.49 (persuasion), 2.5 – 3.49 (use sometimes), and 3.5 – 4.0 (use always)

- = No data available

* SD = NR means standard deviation not reported

** = Only includes nurses who were aware of the practices
